# Language Justice: Addressing Linguistic Disparities Begins with Language Data Collection

**DOI:** 10.4269/ajtmh.23-0237

**Published:** 2023-05-01

**Authors:** Nasreen S. Quadri, Syreeta Wilkins, Kristina Krohn, Erin M. Mann, William M. Stauffer, Patricia F. Walker

**Affiliations:** ^1^ Departments of Medicine, Pediatrics, Infectious Diseases and International Medicine, University of Minnesota, Minneapolis, Minnesota;; ^2^ Center for Global Health and Social Responsibility, National Resource Center for Refugees, Immigrants and Migrants, University of Minnesota, Minneapolis, Minnesota;; ^3^ Allina Health, Minneapolis, Minnesota; ^4^ HealthPartners Institute, Bloomington, Minnesota; ^5^ HealthPartners Travel and Tropical Medicine Center, Saint Paul, Minnesota

The COVID-19 pandemic brought renewed energy and interest to research on health disparities, but the focus has remained primarily on race and ethnicity to categorize inequities. Disparate health outcomes across communities, including those in at-risk refugee, immigrant, and migrant communities, are directly linked to social determinants of health, such as access to and delivery of healthcare.^1^ Using only race and ethnicity to categorize inequities has notable limitations. Namely, race and ethnicity are overly general, combining communities with varied disease risk, distinct cultures, unique languages, and differing exposures to social determinants of health into large ill-defined groups. The official race and ethnicity categories used in the United States include American Indian or Alaska Native, Asian, Black or African American, Native Hawaiian or Other Pacific Islander, and White; and the ethnicity category of Hispanic or Latino.^2^ These classifications do not reflect variation within racial categories based on lived experiences and exposure to social determinants of health. For example, a Hmong refugee may have differing health needs than a Japanese graduate student, yet both would be classified as Asian. While important for measuring systemic racism, race and ethnicity categories are less likely to provide practical information for clinical interventions that improve health outcomes.^3^ In the search for more actionable demographic information for identifying people affected by health inequities, collecting data on the language a patient prefers to speak in a healthcare encounter builds on racial and ethnic categories by addressing linguistic barriers in access to health-related knowledge, care, and resources. People who speak languages other than English face barriers accessing care and receiving quality care, leading to poorer health outcomes.^4^^-^^6^ It is imperative that health equity efforts include language needs as a key measure, which may then inform tailored language services.

Considering language access puts the emphasis on barriers alone, while the concept of language justice uplifts the fundamental rights of people to communicate in their primary language, centering the autonomy of all individuals to speak in their preferred language.^7^ Yael Peled, a language ethicist, advocates for providers to be aware of the critical role of language in shaping patients’ health beliefs and practices around both illness and well-being.^8^ This requires that clinicians recognize the limits of communication when they do not share a language and culture with their patient. It can lead to clinicians passing judgement about a patient’s credibility, and patients misinterpreting terminology the clinician may assume is universal. The resulting dynamic ultimately adversely affects the clinician’s ability to provide high quality healthcare from diagnosis to treatment.^8^ A 2001 survey found Hispanic and Asian patients, when compared to White patients, were more likely to report that their doctor did not listen to everything they said, they did not fully understand their doctor, or they had questions during the visit but did not ask them. The study found communication challenges were even worse for Hispanic and Asian patients when English was not their primary language.^9^ A 2021 focus group survey found that older Latino patients reported negative experiences attributed to their language and accent, which affected both the quality of care they reported receiving and their comfort in seeking healthcare services.^10^ Understanding the importance of both language access and language justice centers the diverse and rich lived experiences of patients who speak languages other than English and is an important step towards providing culturally humble and equitable healthcare.

Language and associated health literacy are powerful social determinants of health.^11^^,^^12^ In this edition of the *Journal*, Steiner et al identify language needs as a domain of inequity affecting COVID-19 vaccination coverage.^13^ The authors emphasize the relative lack of language resources for the 26.5 million United States residents speaking primary languages other than English or Spanish. They found marked disparities in completion of the COVID-19 primary vaccination series by preferred language when comparing 25 of the most common languages represented in their dataset to the control group of Spanish and English speakers. The authors concluded that standardized accurate collection and documentation of self-identified preferred language is imperative. Furthermore, analysis of health disparities by preferred language can support tailoring interventions to meet the needs of specific sub-populations. Their findings were consistent with those of another study during the COVID-19 pandemic, which found that communication in a language other than English was an independent risk factor for infection even after adjusting for social factors such as age, race, and ethnicity.^14^ In addition, a recent large scale study on COVID-19 vaccination and associated clinical outcomes found that patients with a language preference other than English had delays in receiving their first COVID-19 vaccine dose and were twice as likely to be hospitalized or die due to COVID-19.^15^ This study definitively demonstrated that language access was associated with clinical outcomes, resulting in certain language groups suffering from increased morbidity and mortality. Accurate collection and documentation of patient self-reported language preference should be an expected best practice for all research, clinical, and systems level public health activities in the United States. Systems must respond with access to interpreters as well as culturally and linguistically appropriate health interventions. It is also essential that these services are fully funded.

We acknowledge challenges inherent to the call for action to address language needs. Firstly, there are over 7,000 languages recognized in the world. The United States Census Bureau assigns codes for the 1,333 languages spoken in the US and then combines similar languages together into 42 groups based primarily on the representative size of the population in the United States that speaks that language.^16^ For example, groupings exist for Slavic languages (Ukrainian, Bulgarian, Czech); Chinese languages (Mandarin, Cantonese, Taiwanese); Other Native languages of North America (Apache, Cherokee, Lakota, Tohono O’odham, Yupik); and West African languages (Twi, Igbo, Yoruba, Wolof). The large number of spoken languages poses a challenge to healthcare and public health systems to standardize collection of all spoken languages, particularly for language speakers not heavily represented in a given region. An additional challenge includes how to group and analyze the multitude of languages in a meaningful way. For example, Steiner et al opted to group Persian and Farsi together, while Mandarin and Cantonese were not combined.^13^ Quadri et al opted to separate Hmong from Laotian, and Nepali from South Asian languages to emphasize languages spoken by refugee populations in the region represented in the dataset.^15^ The development of recommended best practices or considerations for language analysis can support researchers in designing studies and utilizing research results to ameliorate linguistic disparities. Secondly, multilingual individuals may prefer English despite a different primary language, and this preference may be context dependent. Conversely, a patient may report English proficiency but prefer to speak their primary language in healthcare encounters. Still others may read in one language and speak in another. Individuals may still be influenced by the cultural and linguistic health beliefs of their primary language group despite achieving English proficiency and developing English language preference. Lastly, language data collection alone may prove insufficient to identify healthcare access needs for languages used by different cultures in many geographic regions, such as French, Spanish, or Arabic due to the intimate relationship between language and culture. Language services and health care promotion materials should acknowledge the spectrum of language access needs based on lived experience. Developing partnerships with individuals from diverse linguistic communities can provide meaningful context to create actionable solutions around the intersection of geography, country of birth, culture, and language. A language justice framework for identifying linguistic disparities uplifts the centrality of language in navigating healthcare systems and thus offers an actionable target to redress health inequities.
